# Optimization of *In vivo* Imaging Provides a First Look at Mouse Model of Non-Alcoholic Fatty Liver Disease (NAFLD) Using Intravital Microscopy

**DOI:** 10.3389/fimmu.2019.02988

**Published:** 2020-01-08

**Authors:** Rachelle P. Davis, Bas G. J. Surewaard, Madison Turk, Agostina Carestia, Woo-Yong Lee, Björn Petri, Stefan J. Urbanski, Carla S. Coffin, Craig N. Jenne

**Affiliations:** ^1^Department of Microbiology, Immunology, and Infectious Diseases, University of Calgary, Calgary, AB, Canada; ^2^Snyder Institute for Chronic Diseases, University of Calgary, Calgary, AB, Canada; ^3^Department of Physiology and Pharmacology, University of Calgary, Calgary, AB, Canada; ^4^Department of Pathology and Laboratory Medicine, University of Calgary, Calgary, AB, Canada; ^5^Department of Medicine, Cumming School of Medicine, University of Calgary, Calgary, AB, Canada

**Keywords:** intravital imaging, fatty liver, NAFLD, mice, technique

## Abstract

Non-alcoholic fatty liver disease is a spectrum of liver pathology ranging from simple steatosis to steatohepatitis and can progress to diseases associated with poor outcomes including cirrhosis and hepatocellular carcinoma (HCC). NAFLD research has typically focused on the pathophysiology associated with lipid metabolism, using traditional measures such as histology and serum transaminase assessment; these methods have provided key information regarding NAFLD progression. Although valuable, these techniques are limited in providing further insight into the mechanistic details of inflammation associated with NAFLD. Intravital microscopy (IVM) is an advanced tool that allows for real-time visualization of cellular behavior and interaction in a living animal. Extensive IVM imaging has been conducted in liver, but, in the context of NAFLD, this technique has been regularly avoided due to significant tissue autofluorescence, a phenomenon that is exacerbated with steatosis. Here, we demonstrate that, using multiple imaging platforms and optimization techniques to minimize autofluorescence, IVM in fatty liver is possible. Successful fatty liver intravital imaging provides details on cell trafficking, recruitment, function, and behavior in addition to information about blood flow and vessel dynamics, information which was previously difficult to obtain. As more than 30% of the global population is overweight/obese, there is a significant proportion of the population at risk for NAFLD and complications due to NAFLD (liver decompensation, cirrhosis, HCC). IVM has the potential to elucidate the poorly understood mechanisms surrounding liver inflammation and NAFLD progression and possesses the potential to identify key processes that may be targeted for future therapeutic interventions in NAFLD patients.

## Introduction

Non-alcoholic fatty liver disease (NAFLD) is characterized by a spectrum of pathologies ranging from simple hepatocyte steatosis to non-alcoholic steatohepatitis (NASH) (1). Individuals with NAFLD, and especially those with NASH, are at risk of progression to more significant liver disease such as cirrhosis and hepatocellular carcinoma (2). Typically, NAFLD is associated with a body mass index (BMI) > 35, diabetes, dyslipidemia, and the metabolic syndrome (3). It is estimated that 30% of the North American population is overweight or obese, suggesting a significant proportion of the population is at risk for NAFLD (4). In the United States, NAFLD/NASH has become the leading cause of end-stage liver disease requiring liver transplantation (5). Due to the significant burden of NAFLD/NASH, to both society and to the health care system, there is an urgent need to develop improved pre-clinical animal models for the testing of new therapies as well as improving our understanding of mechanisms of disease pathogenesis. Although the methionine-choline deficient diet (MCD) is a common mouse model of NASH, in many ways it does not accurately recapitulate the chronic microenvironment remodeling that occurs in humans with NAFLD (6). Specifically, MCD models of NAFLD try to replicate disease using a short 2 to 3 week time frame. It is thought this rather “acute” model of liver disease does not accurately recapitulate the chronic tissue remodeling that occurs over a period of decades in humans. Considering this, we have implemented a 20 week high fat diet (HFD) for mice that better models the clinical course of NAFLD development (7).

Although first utilized in the middle of the nineteenth century (8–10), intravital microscopy (IVM) has, over the past two decades, moved to the forefront for tracking cell behavior in real-time, in living animals (11–15). This approach provides remarkable insight into in cell dynamics and *in vivo* disease progression within intact tissues. One of the most studied organs by IVM is the liver. In addition to having a central role in metabolism and protein synthesis, the liver is also an important front-line immune tissue. The liver is crucial for pathogen clearance from the circulation, immunosurveillance, and is a common site for both primary and metastatic cancers (11, 16–19). In fact, more than 800 papers have been published using IVM in the liver (20). Although mouse models of disease have proven extremely useful for studying immune cell interactions in infection and tissue repair (21–23), there remains a distinct gap in the IVM literature with regards to mouse models of NAFLD. The primary reason for this paucity of studies is the notoriously difficult and unique issues involved with imaging a fatty liver. The liver is known to possess a relatively strong profile of autofluorescence, which is greatly exacerbated in conditions of lipid accumulation (24). Due to this significant imaging obstacle, fluorescent IVM of NAFLD in a mouse model has, in the past, been consistently avoided.

Autofluorescence in the liver originates from several sources and can have variable excitation and emission profiles. For example, vitamin A is naturally fluorescent and is found in relatively high concentration in hepatic stellate cells within the liver (25). Similarly, nicotinamide adenine dinucleotide (NADH) fluorescence is common within hepatocytes in the liver (25). Other sources of autofluorescence include metabolites and fatty acids (26). Many of these endogenous emission signals are present in the 400–500 nm range, and thus, the liver exhibits naturally high fluorescence using UV and 488 nm excitation lasers in particular (27). Importantly, this natural autofluorescence is enhanced by the lipid accumulation and metabolic stress that are hallmark in NAFLD. Following significant optimization, and in consideration of the biophotonics of liver imaging, we have developed a successful multicolour IVM approach that enables the study of this tissue in NAFLD mice. This article explains in detail the differences between imaging of fatty liver and healthy controls and delineates the methodological approach to successful image acquisition based on some of the different microscope platforms available.

## Materials and Methods

### Mice and Model of NAFLD

C57Bl/6 mice were purchased from Jackson Laboratories (Bar Harbor, ME). Animals were housed in a pathogen-free environment at the University of Calgary. All experimental protocols were approved by the University of Calgary Animal Care Committee and were in compliance with guidelines from the Canadian Council for Animal Care (AC16-0040; AC18-0050). NAFLD mice were fed a high fat, high sucrose diet obtained from Dyets Inc (Bethlehem, PA) *ad libitum* for 20 weeks beginning at 6 weeks of age. Diet was a custom formula containing 40% fat, 40% sucrose, 20% protein. Control mice were fed standard mouse chow and housed in the same facility to ensure comparable microbiome.

### Antibodies and Reagents

Conjugated antibodies used for *in vivo* imaging are as follows: PE-conjugated anti-CD49b (platelets), Alexa Fluor (AF) 647-conjugated anti-F4/80 (Kupffer cells), Brilliant Violet (BV) 421-conjugated anti-Ly6G (neutrophils), and PerCP/Cy5.5-conjugated anti-CD8a. All antibodies were purchased from Biolegend Inc. (San Diego, CA, USA). Antibodies were administered at a dose of 1–3 μg/animal intravenously (i.v.) 10 min prior to imaging. Polystyrene beads (Fluoresbrite YG microspheres 1.0 μm, Polysciences Inc., Washington, PA, USA) were injected intravenously during image acquisition to track blood flow and to measure Kupffer cell function. Rhodamine-conjugated dextran was used as vascular contrast agent for visualization of liver sinusoids; injected intravenously 10 min prior to imaging.

### Intravital Microscopy

Surgical preparation of animals for intravital microscopy of the mouse liver was performed as previously described (28). After general anesthesia (10 mg/kg xylazine hydrochloride and 200 mg/kg ketamine hydrochloride), an i.v. catheter was inserted in the tail vein to administer fluorescently labeled antibodies or additional anesthetic directly into the bloodstream. For surgery, a laparotomy was performed, and the abdominal skin and peritoneum were removed to expose the liver. The falciform ligament was cut after securing the sternum away from the liver using a suture. The mouse was moved to a heated stage, to maintain body temperature throughout image acquisition, and placed on its right side. Using a wet cotton swab, the stomach was manipulated to maneuver the liver into place on a glass coverslip. The gastrointestinal tract was moved away from the liver and secured by wrapping in wet gauze. One layer of wet tissue was placed on the liver to preserve physiological conditions, prevent drying, and diminish movement. Imaging was performed using an inverted Leica SP8 resonance-scanning microscope (Leica Microsystems) with a 25× water-immersion objective lens. For spinning-disk microscopy an inverted microscope (IX81; Olympus) was used, equipped with a focus drive (Olympus), a motorized stage (Applied Scientific Instrumentation), and fitted with a motorized objective turret equipped with 20×/0.70 UPLANSAPO objective lenses coupled to a confocal light path (WaveFx; Quorum Technologies) based on a modified CSU-10 head (Yokogawa Electric Corporation).

### IVM Analysis

For vessel diameter, sinusoids were manually measured using the LasX software ruler. Each FOV had 10 sinusoidal measurements to accurately represent the entire FOV. For cell/particle quantification, random fields of view (FOV) were chosen and the number of events were counted for a given frame. For blood flow velocity, sinusoids between 80 and 120 μm of length were identified and the velocity of fluorescent beads passing through these sinusoids was measured. For each bead, a time measurement in seconds to traverse the measured distance was recorded. Distance was divided by time, and the measurements were repeated in a minimum of five sinusoids/FOV.

$$\begin{array}{l} Blood\;Flow\;Velocity\;\left(\frac{\mu m}{s}\right)=\;\frac{distance\;bead\;traveled\;in\;\mu m\;}{time\;required\;to\;travese\;measure\;distance} \end{array}$$

To calculate flow rate, sinusoidal measurements of vascular diameter allowed for the calculation of the sinusoidal cross-section area. Blood velocity (from the bead calculations) was applied to determine the overall flow rate in μm^3^/s.

$$\begin{array}{l} Blood\;Flow\;Rate\;\left(\frac{{\mu m}^{3}}{s}\right)=\;\pi *\;sinusoidal\;{radius\;\left(\mu m\right)}^{2}\\ \;*\;blood\;flow\;velocity\;\left(\frac{\mu m}{s}\right) \end{array}$$

Cell behaviors as visualized by IVM were characterized as either stationary (cells that move <1 cell diameter in 3 min of imaging) or crawling (cells interacting with the endothelium and traversing >1 cell diameter in 3 min).

### Histology and Alanine Aminotransferase (ALT) Measurements

Following euthanasia, liver samples were collected and fixed in formalin. Paraffin embedding was performed, and 4.0 μm sections were stained with hematoxylin and eosin (H&E). All mouse slides were assessed by a blinded hepatopathologist to determine the degree of steatosis, the presence of inflammation and hepatocyte ballooning. Blood was collected via cardiac puncture using a 1:10 dilution of 4% sodium citrate (Sigma Aldrich), and then centrifuged at 1,000 × g for 10 min to obtain plasma. Blood plasma samples were sent to Calgary Lab Services (CLS, Calgary, Canada) and ALT measurements were performed to assess degree of liver tissue damage.

### Progressive Emission Filter Scan (Lambda Scan)

Marked autofluorescence can be observed in tissues depending on the excitation/emission spectra used. To assemble a complete fluorescence profile of the liver, the Lambda Scan feature of LasX software (Leica microsystems) was utilized. Fixed excitation wavelength lasers were used, one at a time, and emission filters positioned in front of HyD detectors were set to function as 50 nm-wide band pass filters. Scanning was performed using 20 nm step-wise advancements of the emission filter via automated algorithm; settings were as follows: for the 405 nm excitation laser (50% power), emission filters scanned from 425 to 775 nm; for the 488 nm excitation laser (10% power), emission filters scanned from 510 to 760 nm; for the 552 nm excitation laser (10% power), emission filters scanned from 560 to 770 nm; and for the 638 nm excitation laser (5% power), emission filters scanned from 645 to 775 nm. Snapshots for individual excitation wavelengths were captured at each 20 nm interval within the scan range.

### Statistical Analysis

With the exception of velocity measurements, a Student's *T*-test was used to determine statistical significance in all instances. For comparison of velocity measurements, a One-way ANOVA with Tukey post-test was used. All reported measurements represent the mean of 5 FOV/animal. *N* = 5 animals/group, ^*^p < 0.05, ^**^p < 0.01, ^***^p < 0.001.

## Results

### 20 Week HFD Generates a Reproducible NAFLD Phenotype With Elevated Autofluorescence

**Figure 1:**
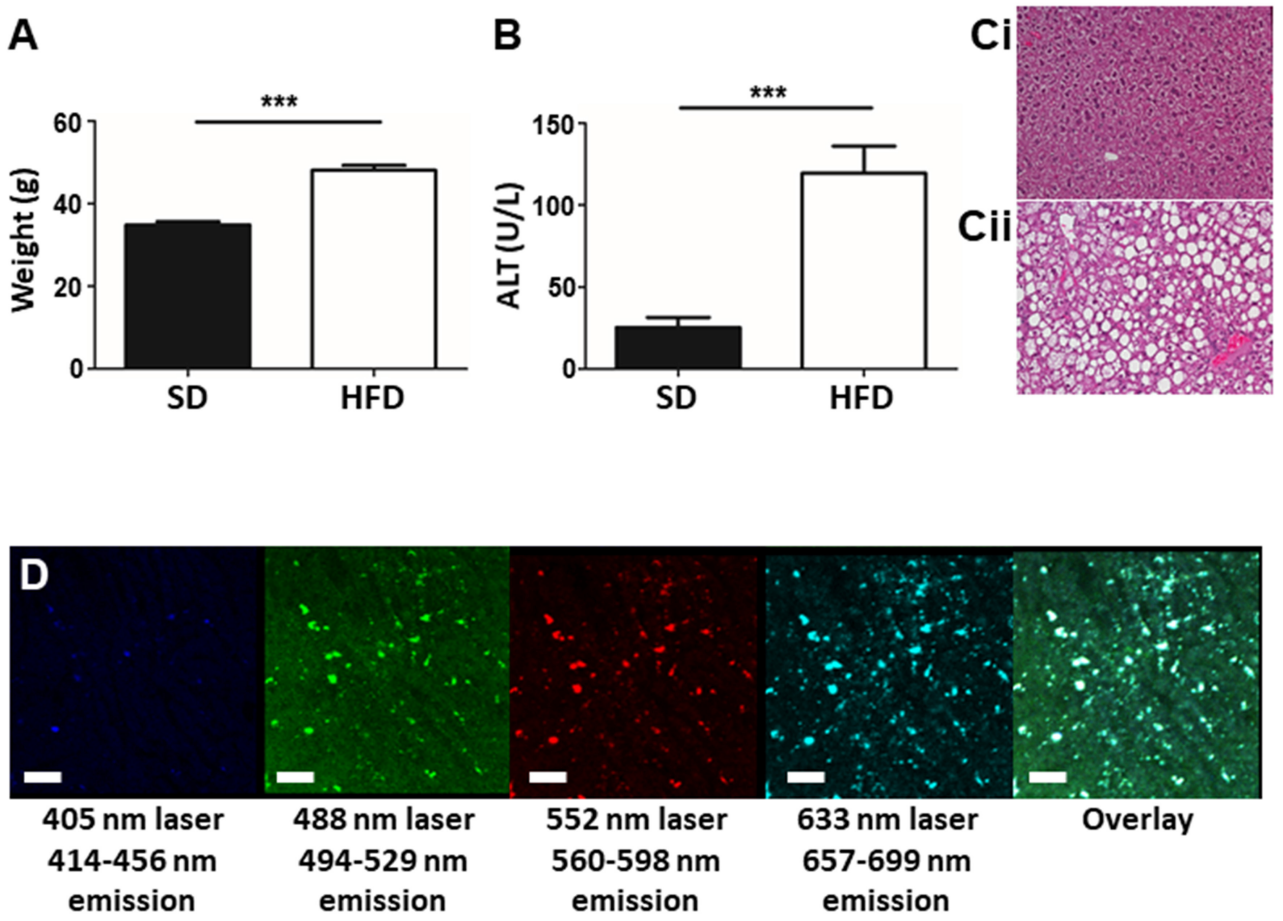
Establishment of a mouse model of NAFLD. Animals were fed either SD or HFD for 20 w and animal weight **(A)** and plasma ALT levels **(B)** were measured. Histology of the mouse liver reveals normal tissue architecture in animals fed SD **(Ci)**, whereas, animals fed HFD **(Cii)** display pronounced steatosis and hepatocyte ballooning. IVM imaging of unstained liver (i.e., no exogenous fluorophores or dyes added) shows an overall autofluorescence dotted by focal intense spots of fluorescence (white spots in the overlay image) (scale bar = 100 μm) **(D)**. ^***^p < 0.001.

To determine the liver phenotype following HFD for 20 weeks, animal body mass, liver damage, and lipid accumulation was assessed in mice fed HFD and in age-matched controls. Mice on HFD had significantly increased body mass compared to age-matched control animals (Figure 1A). Plasma ALT levels indicated significant hepatic damage in HFD mice compared to controls (Figure 1B) and histological analysis of the liver revealed marked lipid deposition within hepatocytes of HFD mice, whereas minimal steatosis was observed in controls (Figure 1C). Overall, mice fed an HFD show a consistent NAFLD phenotype, involving weight gain, steatosis, and elevated ALT levels validating the selected mouse model of NAFLD. The steatosis observed in mice fed HFD resulted in significant liver autofluorescence in a wide range of wavelengths when imaged by IVM (Figure 1D). Even in the absence of added fluorescent labels or dyes, strong background autofluorescence dotted with foci of high intensity signal are seen throughout the liver, in multiple emission wavelengths.

### Lambda Scanning for Autofluorescent Profile Mapping

As illustrated in Figure 1D, lipid dense tissues demonstrate significant autofluorescence when visualized by confocal microscopy. To better map the specific wavelengths of light attributed to this autofluorescent signal a spectral scan was performed. A spectral scan involves imaging a given tissue with a fixed excitation wavelength and a narrow, step-wise shifting emission filter. In a scan, a single excitation laser is used, and fluorescence is captured for each of a series of sequential detection filter ranges. For example, fluorescence is captured through a series of sequential, overlapping 50 nm gates (400–450, 420–470, 440–490 nm, etc.) With this approach, we mapped the complete autofluorescent signature of the liver for each excitation laser (Supplemental Figure 1).

Mapping the autofluorescence profile in this manner is informative regarding which specific fluorophores should be avoided (emission overlap with regions of high autofluorescence), and which fluorophores may be better options. With respect to the Leica SP8 imaging platform, spectral mapping can be done through an automatic feature, the Lambda Scan, where both the emission detector bandwidth and number of imaging steps can be specified. Lambda scanning was performed on NAFLD mice to map the full autofluorescence profile. Results show substantial autofluorescence between 450 and 520 nm when excited by the 405 nm laser and between 550 and 650 nm when excited by the 488 nm laser. Additionally, discrete, punctate autofluorescence can been seen in most imaging windows. We utilized this spectral scan to choose antibodies: those that have a higher Stokes shift (maximal separation of excitation and emission spectra of a given fluorophore) and are excited outside of the 488/552 nm range to avoid the autofluorescent characteristics of fatty liver.

### IVM Image Acquisition: Optimization for NAFLD and Microscope Platform

To minimize the autofluorescence phenomenon, and to ensure it was possible to identify and track multiple cell populations within a fatty liver, we employed several optimization strategies. First, microscope settings were selected to utilize sequential scanning. This approach involves cycling each excitation laser on and off in a sequence, to minimize the collection of non-specific light (background) from sub-optimal excitation (i.e., excite with single wavelengths sequentially as opposed to multiple excitation wavelengths simultaneously). Often, multiple laser lines are switched on together, allowing for simultaneous excitation and visualization of multiple fluorophores by different optical detectors. Although this approach supports rapid imaging of samples, it can generate unwanted fluorescence through off-peak excitation of fluorescent compounds or molecules. For example, molecules that are normally not excited by a 488 nm laser may be excited by a 405 nm laser and bleed into photodetectors used to image fluorophores excited by the 488 nm laser. In this case, ensuring the 405 nm laser is off when imaging with the 488 nm laser will limit excitation and bleed through of unintended fluorescent signals.

**Figure 2:**
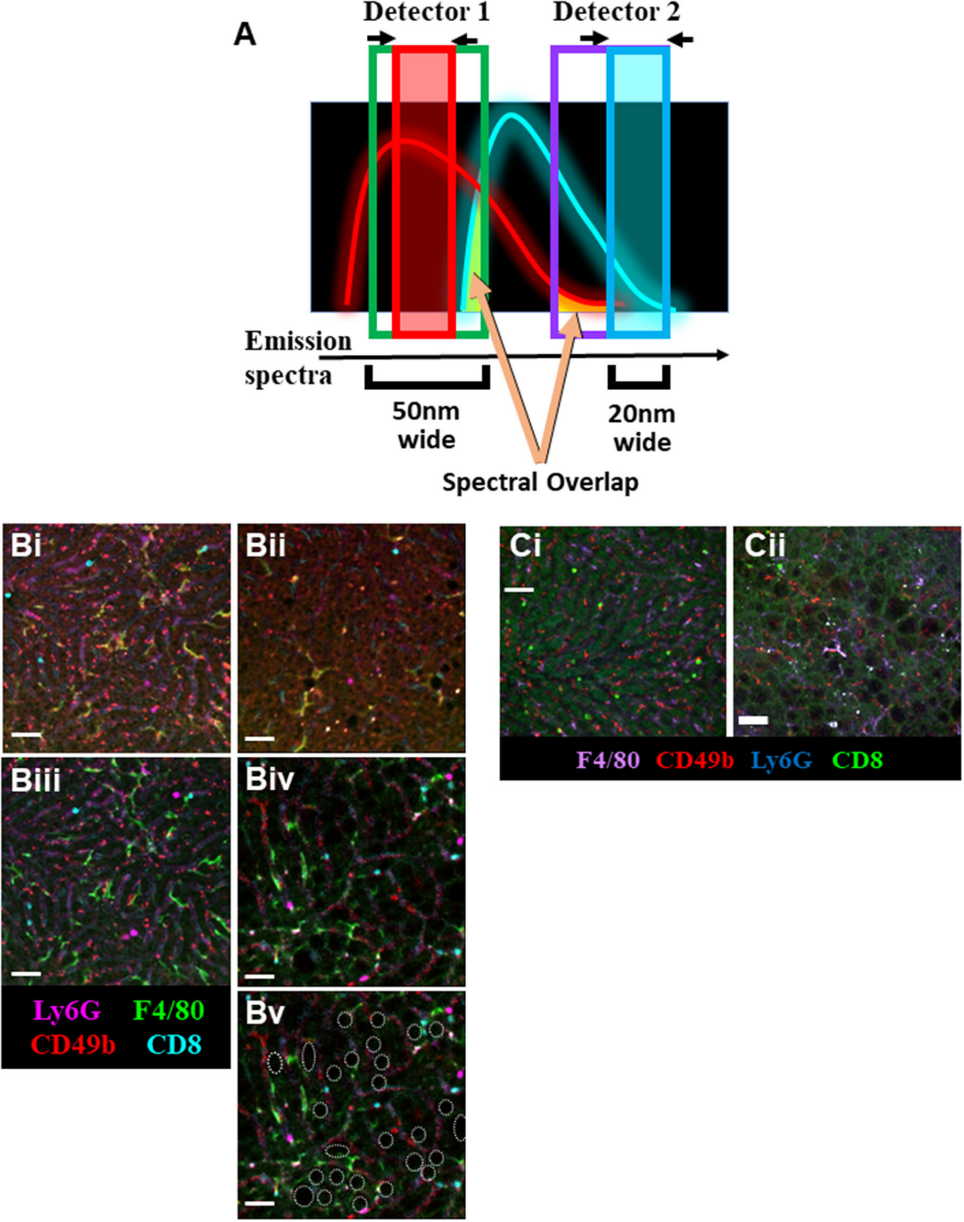
Optimization of IVM of murine NAFLD. **(A)** Schematic representation of the imaging strategy utilizing narrower emission filters (red and blue boxes) that have been shifted away from peak fluorophore emission to limit the collection of non-specific spectral overlap (yellow shading). IVM of liver using resonant scanning confocal microscopy with conventional filter settings in animals fed SD **(Bi)** or HFD **(Bii)**. RSC imaging of livers from mice fed SD **(Biii)** or HFD **(Biv)** using optimized (narrowed filters, off-peak fluorescence collection, sequential excitation) imaging parameters yields substantially less background autofluorescence and allows for clear visualization of multiple labeled immune cell populations. Cell populations were labeled with i.v. fluorophore-conjugated antibodies 10 min prior to imaging (scale bar = 50 μm). Additionally, lipid deposits are visible in the liver of mice fed HFD (**Bv**, white outlines; same FOV as **Biv**). IVM of liver using spinning disk microscopy in animals fed SD **(Ci)** or HFD **(Cii)**. Animals were i.v. injected with fluorophore-conjugated antibodies 10 min prior to imaging (scale bar = 50 μm).

Additionally, the use of a tunable Spectral Detector emission filter located before the optical detectors [photomultiplier tubes (PMTs) or hybrid gapless detectors (HyDs)] allowed us to narrow the wavelength range of the captured emission fluorescence (Figure 2A). This process allows us to maximize detection of desired emission spectra while “cropping-out” non-specific autofluorescence spectral overlap signal. Although narrowing the emission filter captures less light, this is outweighed by the advantage of excluding background and spectral overlap from other fluorophores.

To compensate for the loss of collected fluorescent signal resulting from the use of narrow emission filters, other settings required adjustment to compensate. The resonance scanning confocal microscope (RSM) platform used in these experiments was equipped with a mix of PMTs and HyDs for signal detection. Whereas, PMTs are excellent at imaging bright fluorophores or fluorophores with emissions below 400 nm or above 750 nm, they often require high excitation laser power and substantial signal amplification (gain) at the detector (Supplemental Table 1). In contrast, HyD-based detectors are significantly more sensitive, allowing for detection of dimmer signals with less electronic amplification (thus less signal noise), ideal for imaging through narrow wavelength emission filters. Additionally, using an RSM allowed for the adjustments to be made to the optical pinhole diameter, increasing photon capture. Normally, a smaller pinhole allows for the capture of less out-of-focus light rendering a higher resolution image; however, opening the pinhole slightly maximizes light capture with minimal sacrifice of image resolution. Pinhole adjustments are possible and straightforward on most scanning confocal imaging platforms. In contrast, although possible, adjusting the pinhole diameter on most spinning-disk microscope platforms is not simple and often requires additional hardware installation (29). Optimal imaging of the autofluorescent liver requires balance of all these settings; therefore, careful consideration of fluorophore combinations is needed in order to acquire the best image possible with minimal background.

The livers of mice fed a SD can be effectually and clearly imaged utilizing conventional settings (Supplemental Table 2) on an RSM (Figure 2Bi); however, imaging of cellular targets labeled with fluorophore-conjugated antibodies is nearly impossible in the liver of mice fed an HFD (Figure 2Bii). Although application of sequential imaging, narrow emission filters, and optimized pinhole can enhance imaging of livers in SD mice (Figure 2Biii), this approach is more critical in HFD mice where specific fluorophore signals that could not previously be separated from autofluorescence are now imaged as discrete, discernable markers (Figure 2Biv). This approach not only allowed for clear visualization of specific labels, but also highlighted anatomical changes in the liver with lipid deposits now easily identifiable as dark circular structures located between the liver sinusoids (outlined in white, Figure 2Bv).

Following optimization of fatty liver imaging on an RSM platform, we next compared image quality to that obtained on a spinning-disk microscope (fixed pinhole diameter, non-tunable emission detectors, camera-based images as compared to PMT/HyD-based imaging on RSM). It should be noted that although our spinning-disk microscope utilizes multiple excitation laser wavelengths, these laser lines are not identical to the RSM platform (491, 561, and 642 nm for SDM vs. 405, 488, 552, and 638 nm for RSM). This makes use of the identical labeling antibodies difficult, and thus comparison of specific markers is not optimal; however, general comparison of background fluorescence and the separation of signal from noise/background is possible. Imaging healthy livers (SD) using a spinning-disk microscope yields clear images, capable of discerning multiple fluorescent markers and tracking several cell types in real-time (Figure 2Ci). This imaging is very comparable to what is captured in SD mice using the optimized RSM protocol (Figure 2Bii). Importantly, when imaging the liver of mice fed a HFD, spinning-disk microscopy demonstrates limitations (Figure 2Cii). Due to the fixed (glass) band-pass filters located in front of the image capture CCD camera, it is difficult to optimize imaging windows to enhance signal collection while limiting non-specific spectral bleed-through. In much the same fashion as the non-optimized RSM (Figure 2Bii), spinning-disk microscopy of liver in HFD animals captures numerous patches of generalized autofluorescence and a punctate pattern of high intensity signal in multiple channels (appearing as white spots). In contrast, optimized RSM of HFD livers reduces the diffuse autofluorescence and eliminates the high intensity autofluorescent areas (Figure 2Biv), allowing for tracking of several cell types within this diseased liver.

### IVM to Visualize Liver Physiology and Cell Tracking

**Figure 3:**
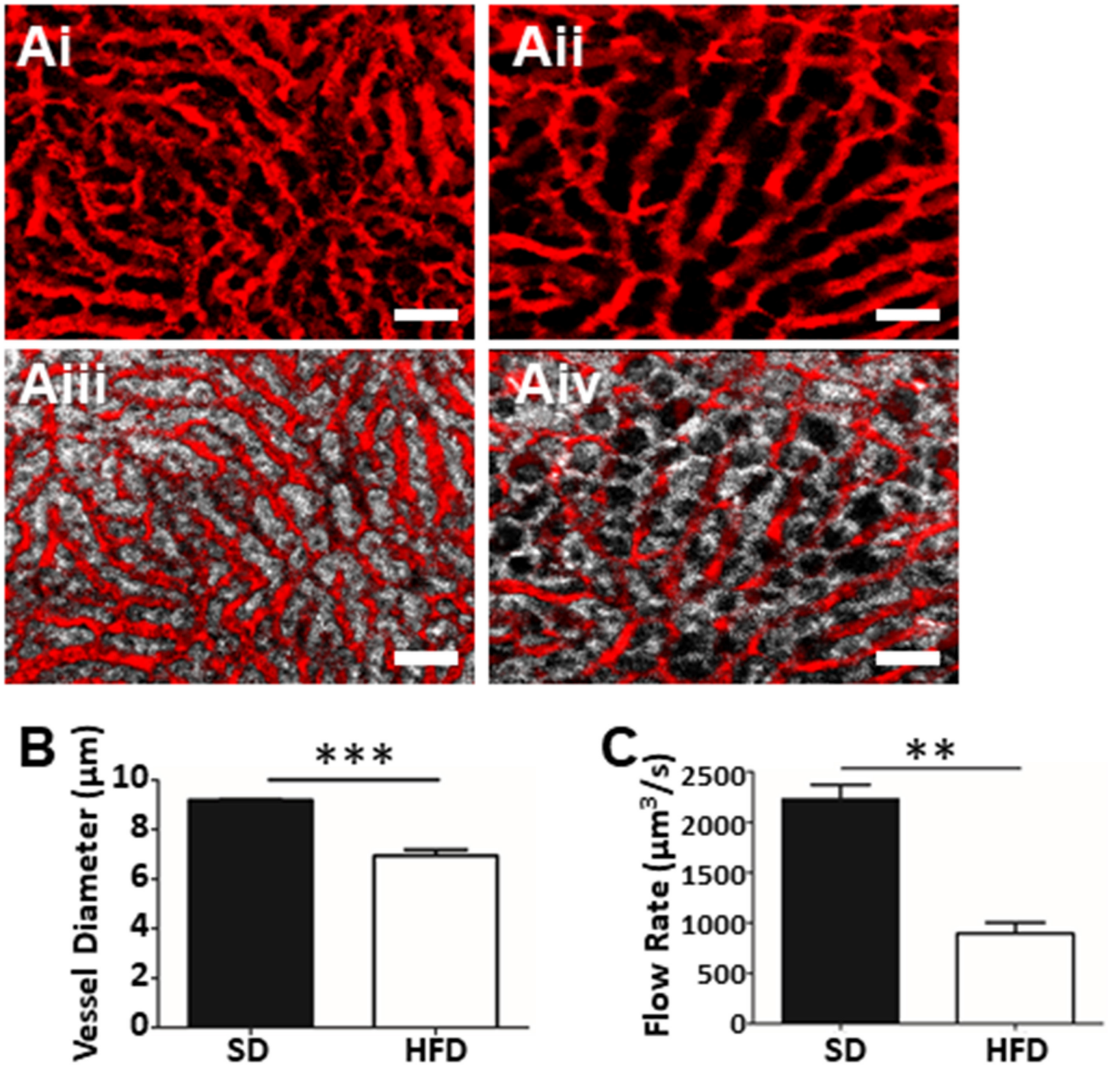
Assessment of fatty liver physiology using IVM. Mapping of liver vasculature in mice fed SD **(Ai)** or HFD **(Aii)** using an i.v. injection of FITC-conjugated albumin (red). Overlay of hepatocyte fluorescence with vascular contrast agent (SD, **Aiii**; HFD, **Aiv**) (scale bar = 50 μm). **(B)** Mean liver sinusoidal diameter as measured by IVM (values represent vascular diameter in μm, mean of >10 sinusoids/FOV; 5 FOV/animal, *n* = 3 animals per group). Mean sinusoidal blood flow rate as determined by IVM **(C)**. Rate was calculated by measuring the sinusoidal cross-sectional area and blood velocity (as determined by velocity of i.v. injected fluorescent beads) and values are reported as μm^3^/s. ^***^p < 0.001; ^**^p < 0.01.

The greatest advantage of IVM over other techniques (histology, FACS, etc.) is the ability to assess intact tissues under physiological conditions. Intravenous delivery of fluorophore-conjugated albumin rapidly highlights the vasculature, permitting the identification, and tracing of the living network of liver sinusoids and venules. The use of this vascular contrast agent in mice fed SD illustrates a dense honeycomb of liver sinusoids that converge on larger draining venules (Figure 3Ai). This vasculature is interwoven with clear columns of hepatocytes separating the sinusoids (Figure 3Aiii). In contrast, the liver vasculature of mice fed a HFD (Figures 3Aii,iv) demonstrates a restricted and convoluted network that bends and wraps around the hepatocyte lipid deposits (dark circular voids in Figure 3Aiv) associated with the steatosis seen in NAFLD. Direct measurement of the images allowed us to determine vascular diameter (Figure 3B). Assessment of multiple vessels per field of view (FOV), and multiple FOVs per animal provided an overall picture of vascular physiology and demonstrates statistically narrower liver sinusoids in the HFD mice. Furthermore, by introducing fluorescently-conjugated antibodies to label platelets, or i.v. injection of fluorescent polystyrene beads (1 μm in diameter), we were able to determine the blood flow velocity within the liver sinusoids. Combining this velocity, with vascular diameters allows for the calculation the overall hepatic blood flow (Figure 3C). Not surprisingly, given the restriction in vascular diameter, animals fed HFD demonstrated significantly reduced blood flow through the hepatic sinusoids when compared to blood flow in the livers of healthy mice.

**Figure 4:**
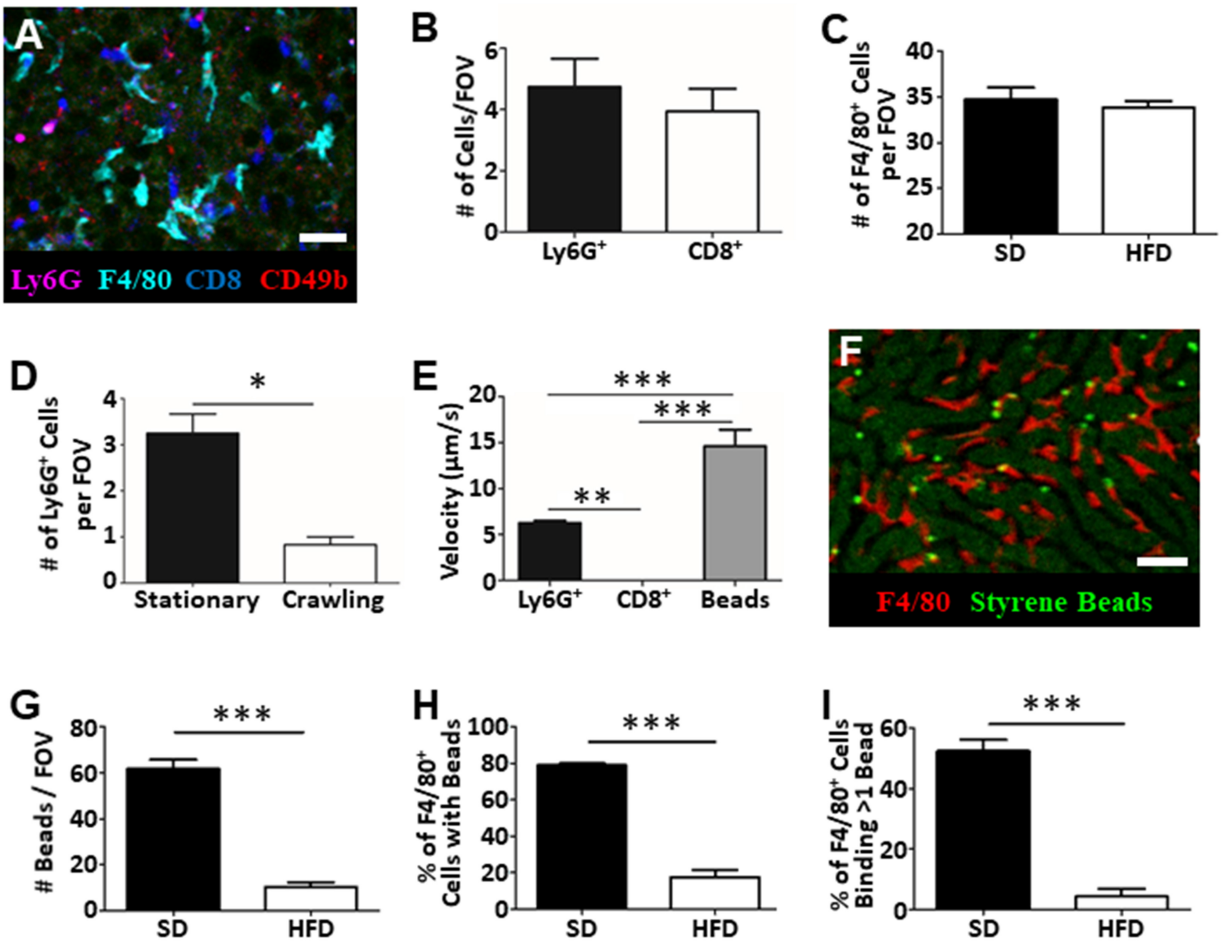
Tracking of immune cell behavior and function in fatty liver by IVM. Fluorescent antibody labeling of multiple immune cell populations enables tracking of cell behavior and function by IVM **(A)**. Cells were labeled by i.v. injection fluorophore-conjugated antibodies 10 min prior to imaging. Scale bar = 50 μm. **(B)** Quantification of the number of neutrophils (Ly6G+) and cytotoxic T cells (CD8+) by IVM in the livers of mice fed HFD (values represent the number of cells/FOV; minimum of 5 FOV/animal; *n* = 3 animals). **(C)** Quantification of the number of macrophage (F4/80+) by IVM in the livers of mice fed SD or HFD (values represent the number of cells/FOV; minimum of 5 FOV/animal; *n* = 3 animals per group). **(D)** IVM assessment of neutrophil behavior in the livers of mice HFD; 5 FOV/animal; *n* = 3 animals. **(E)** Determination of neutrophil (Ly6G+), cytotoxic T cell (CD8+) or intravascular bead velocity in the livers of mice fed HFD (values represent mean object velocity in μm/s from 5 FOV/animal; *n* = 3 animals). **(F)** Representative image obtained by IVM of sterile bead capture (bright green) by liver macrophage (F4/80+; red). Scale bar = 50 μm. **(G)** Quantification of bead capture in the liver (values represent the number of beads/FOV; minimum of 5 FOV/animal; *n* = 3 animals). **(H)** Determination of the fraction of macrophage that have captured a minimum of 1 bead (values represent the % of macrophages binding beads/FOV; minimum of 5 FOV/animal; *n* = 3 animals). **(I)** Determination of fraction of macrophage that have bound multiple beads (values represent the % of macrophages binding multiple beads/FOV; minimum of 5 FOV/animal; *n* = 3 animals). ^***^p < 0.001; ^**^p < 0.01; ^*^p < 0.05.

IVM is not restricted to assessing vascular function and blood flow dynamics in the liver. Intravenous introduction of fluorophore-conjugated antibodies against specific cell surface markers, or the use of mice expressing fluorescent reporter proteins, allows specific cell populations to be labeled and tracked in real-time. Through the addition of anti-Ly6G (labels neutrophils), anti-F4/80 (labels macrophage), anti-CD8 (labels cytotoxic T cells), and anti-CD49b (labels platelets and NK cells) we were able to visualize and track multiple cell populations in simultaneously in the living animal (Figure 4A) (14, 17, 21, 23). With this approach we directly enumerated the number of cells visible per FOV (Figures 4B,C) and characterized cellular behavior (Figures 4D,E). For example, tracking of neutrophils within the liver vasculature revealed significantly more stationary cells than crawling cells in the fatty liver (Figure 4D). This observation is consistent with what has been observed in livers of healthy mice where few actively crawling cells are seen (13, 30).

Moreover, as these images and associated videos are collected in real time, we are able measure the velocity of the crawling cells and the velocity of circulating fluorescent beads (Figure 4E). In this instance, we have determined that neutrophils move significantly faster than CD8+ T cells but are significantly slower than objects (beads) observed freely circulating in the bloodstream. One of the primary immune functions of the liver is to filter circulating pathogens from the blood; a process that is largely mediated by the liver-resident macrophage population, the Kupffer cells (KC) (31, 32). Through the introduction of small (1 μm), sterile, fluorescent polystyrene beads into the circulation, we were able to visualize KC binding and capture of the circulating microspheres (Figure 4F). Comparison of target capture in healthy mice and mice fed an HFD demonstrated a reduced capacity for pathogen clearance in the fatty liver (Figure 4G) as reflected by a significant reduction in bead capture. Importantly, because we were able to directly visualize these immune responses, we determined that the observed deficiency in bead capture stems from both a reduced number of KCs “catching” beads (Figure 4H) and, a reduced capacity, on a per cell basis, to capture multiple targets (Figure 4I). These basic analyses demonstrate the power of IVM to assess liver physiology and immune function within a living, intact tissue and offer the ability to shed light on key processes (immune cell recruitment, cell behavior, cell-cell interactions, vascular tone, and blood flow) associated with disease progression and liver dysfunction within the context of fatty liver disease.

## Discussion

In the current study, we demonstrate how IVM can be used to study liver physiology and immune cell behavior in a murine model of NAFLD in animals receiving long-term HFD. To our knowledge, this technique for imaging NAFLD has not been reported to date. Optimization of multi-color imaging provides a new approach to study NAFLD in a living animal in real-time. Although many different murine models of NAFLD exist, each with their own advantages/disadvantages, the model chosen for this study involves a 20 week HFD. This model not only recapitulates the key clinical features of NAFLD (based on histology and serology), but also allows adequate time for critical immune and tissue reprogramming (macrophage phenotypes, T cell subsets) and remodeling (vascular structure, fibrosis). This reprogramming is critically important when one considers the diverse population of liver resident immune cell populations and phenotypes (Kupffer cells, *i*NKT cells, NK cells, etc.). We believe this chronic model is a more accurate depiction of human disease progression, a condition that takes decades to develop in patients, affording time for immune cell populations to reprogram to respond to increased hepatocyte stress and tissue remodeling.

For more than two decades, IVM has been used to study liver biology and immune responses. Utilizing technologies ranging from white light microscopy to epifluorescence, confocal microscopy (spinning disk, laser scanning, resonant scanning) and multiphoton microscopy, we have gained much knowledge of liver physiology, inflammation, tissue repair, and host-pathogen interactions (23, 33–38). Although these approaches have generated a wealth of information by mapping cell behaviors, cell-cell interactions and generating detailed 3D renderings of liver anatomy, most studies have only looked at healthy livers or at tissues following acute challenge (infection, sterile injury). Liver conditions such as NAFLD, where there is substantial accumulation of lipid within the liver and critical changes in mitochondrial physiology (39, 40), often alter the fluorescent properties of the liver tissue itself. Although this has little impact on techniques such as white light microscopy, the impact on fluorescent imaging is far more significant. As we have demonstrated, NAFLD results in increased autofluorescence within the diseased liver, which makes the tracking of fluorescently labeled targets quite difficult with standard imaging approaches. Through the use of advanced microscope technologies, technologies that in recent years have become accessible and available to many research institutions, and careful optimization of intravital imaging parameters, it is now possible to track multiple populations of individual immune cells within a mouse model of NAFLD. It should be noted that imaging strategies described herein have been optimized on a Leica SP8 microscope. Although overall optimization approaches are applicable across multiple platforms, specific settings (laser power, detector sensitivity/exposure time, pinholes, etc.) will likely have to be empirically determined on each individual imaging platform for best performance.

This new imaging capability opens important doors in the area of NAFLD research, allowing us to address a myriad of different questions that are simply not possible with other techniques; How does a fatty liver respond to pathogen challenge? How do resident immune cells respond to potential pharmacological therapeutics in a fatty liver? How do leukocyte populations interact with each other and with the liver parenchyma? By visualizing tissue physiology, blood flow, pathogen interactions, and cell behaviors within intact living tissues, in real-time, these questions can now be answered. For example, multiple studies have noted an increase in macrophage or decrease in CD4+ T cell populations in the liver during the development of NAFLD but these studies have not completely determined how these cell populations interact within the liver microenvironment nor have they fully mapped how these populations contribute to disease progression (41–43). Given the central role the immune system plays in the progression of liver disease, results in this context will be critical to advance understanding of disease pathogenesis as well as therapeutic interventions for patients with NAFLD.

Due to the elevated autofluorescence found in a fatty liver, accurate real-time imaging has been difficult to achieve (24). RSM provides an attractive platform due its wide range of possible image acquisition settings, highlighting the effectiveness of this technology as a suitable platform for IVM imaging in NAFLD. Further, features such as the Lambda Scan can provide additional detail on spectral regions of fluorescence that can be avoided prior to the development of labeling panels and further image optimization. In fact, this approach of spectral Lambda Scanning and emission filter shifting/optimization could be applied to various types of tissue known to exhibit significant autofluorescence to gain the same insight, and image quality (Supplemental Figure 2). The experimental approaches and data presented in this study provides detailed step-by-step information to optimize IVM imaging in any tissue with marked background fluorescence and provides important considerations to be included in model development by microscopists in both beginner and advanced stages of training. Importantly, IVM is not limited to RSM or spinning-disk microscopy. Additional imaging platforms including widefield fluorescence and two-photon microscopy each have their own advantages and disadvantages with respect to IVM of the liver. For example, two-photon imaging does not require a pinhole, typically results in reduced photodamage, generates less out-of-focus fluorescence and often has higher sensitivity for some fluorophores (20). We did not explore the feasibility of this platform in the imaging of fatty liver in the current work. A recent study has suggested application of this platform for imaging fixed murine fatty liver tissue samples is possible (44); however, an extensive workup of autofluorescence profiles, optimization for imaging multiple cell populations, and application to live animals remains to be completed.

Although this study has demonstrated the power of IVM, there are limitations that should be acknowledged. First, IVM can be restricted in the number of cell types that can be labeled at one time. Typically, the number of colors used is anywhere from one to five, but some studies have visualized up to eight markers in a single liver. This limitation in the number of markers that can be imaged means that observations are often simplified and cannot distinguish specific cell subsets in the same way flow cytometry can. Additionally, many markers used for flow cytometry simply are not bright enough *in vivo* or are based on intracellular targets requiring cell permeabilization prior to labeling making these markers not suitable for IVM. Furthermore, this study did not include the use of multiphoton imaging, a technique that has become increasingly common due to its longer wavelengths allowing for greater tissue penetration and often less autofluorescence.

The obesity epidemic is staggeringly prominent in North America, and NAFLD is quickly becoming the number one indication for liver transplant (45, 46). Considering this, it becomes imperative to delineate the mechanisms associated with the inflammatory progression of NAFLD and provide new techniques to intimately examine the liver microenvironment in the context of NAFLD. We believe the data presented here provides critical new avenues for NAFLD research and represents an exciting new platform for exploration and discovery, especially in the context of immunity. Further experiments using IVM in a mouse model of NAFLD can expose crucial differences in leukocyte trafficking and behavior that may be altered in a NAFLD setting and can provide greater detail into immune mediated fibrosis and functional immune differences that may exist within NAFLD.

## Data Availability Statement

The datasets generated for this study are available on request to the corresponding author.

## Ethics Statement

The animal study was reviewed and approved by University of Calgary Animal Care Committee in compliance with guidelines from the Canadian Council for Animal Care.

## Author Contributions

RD and CJ designed the experiments and wrote the manuscript. CC contributed to experimental design and revised the manuscript. BS, AC, W-YL, and BP contributed to imaging and microscope optimization. MT contributed to imaging and manuscript revision. SU characterized liver pathology and verified the mouse model of NALFD.

### Conflict of Interest

The authors declare that the research was conducted in the absence of any commercial or financial relationships that could be construed as a potential conflict of interest.
