# Supplementary material for: Optimization of In vivo Imaging Provides a First Look at Mouse Model of Non-Alcoholic Fatty Liver Disease (NAFLD) Using Intravital Microscopy
Source: Front Immunol. 2020 Jan 8;10:2988. doi: 10.3389/fimmu.2019.02988 (PMC6960139; doi:10.3389/fimmu.2019.02988)
Supplement: Supplementary file 1 [file Data_Sheet_1.PDF]

Supplementary Material

Supplementary Table 1: Photodetector Choice

Properties	PMT	HyD
Fluorophores	"Bright" (reporter proteins, Alexa Fluor dyes)	"Dim" (broad array of fluorophores, dyes and autofluorescence)
Detector Gain	High (increased noise)	Low (minimal noise)
Laser Power	High (risk of tissue damage, photobleaching)	Low (longer, sustained imaging)
Optimal Wavelengths	Wide spectrum	Lower efficiency at extreme limits (<400, >750nm)
Overall	Better for transgenic reporter proteins or bright fluorophores	Better for dim fluorophores, photobleaching sensitive labels, long-term imaging,

Supplementary Table 2: Optimized Intravital Microscope Filter Settings for NAFLD

Laser Line	Example Fluorophore	Standard Emission Filter Setting	NAFLD Optimized Filter Setting
405 nm	DAPI<br>Brilliant Violet 421	PMT<br>416-452 nm	PMT<br>431-451 nm
488 nm	FITC /<br>Alexa Fluor 488	HyD<br>500-552 nm	HyD<br>498-529 nm
488 nm	Percp-Cy5.5	HyD<br>687-728 nm	HyD<br>694-720 nm
552 nm	Phycoerythrin (PE)<br>Alexa Fluor 595	PMT<br>587-618 nm	PMT<br>577-600 nm
647 nm	Allophycocyanin (APC)<br>Alexa Fluor 647	HyD<br>647-681 nm	HyD<br>647-670 nm

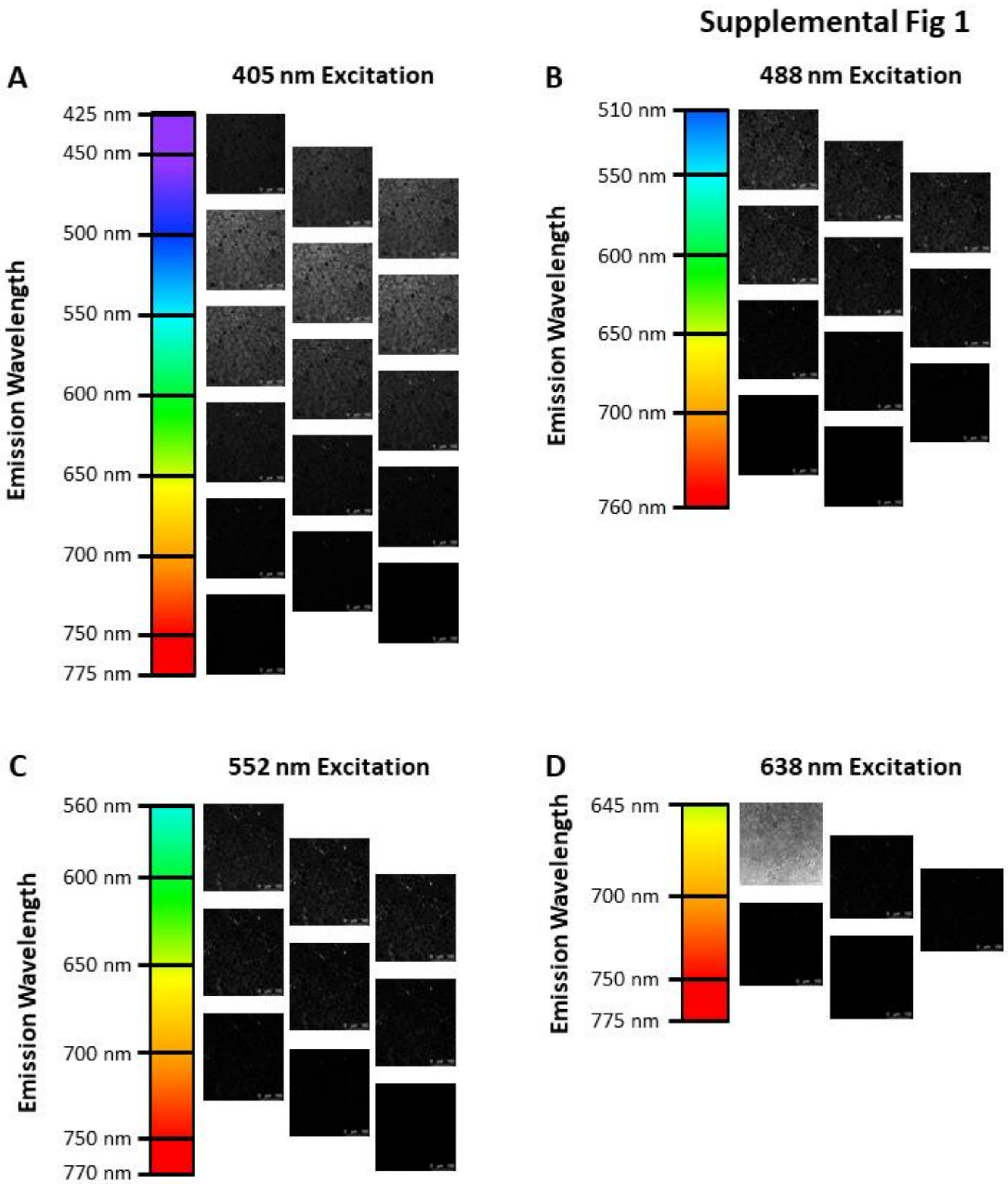

Supplementary Figure 1. Lambda scan of liver from HFD mice illustrating autofluorescence footprint. Representative images of spectral scan of an HFD liver. Scan conducted with a 50nm wide detector imaging step-wise every 20nm along the emission spectrum. Individual scan profiles corresponding to excitation by a 405 nm (**A;** scanning from 425-475 nm emission), a 488 nm (**B;** scanning from 510-760 nm emission), a 552 nm (**C;** scanning from 560-770 nm emission) or a 638 nm (**D;** scanning from 645-775 nm emission) laser. Individual images are scaled and positioned to correspond to the emission range captured for each frame.

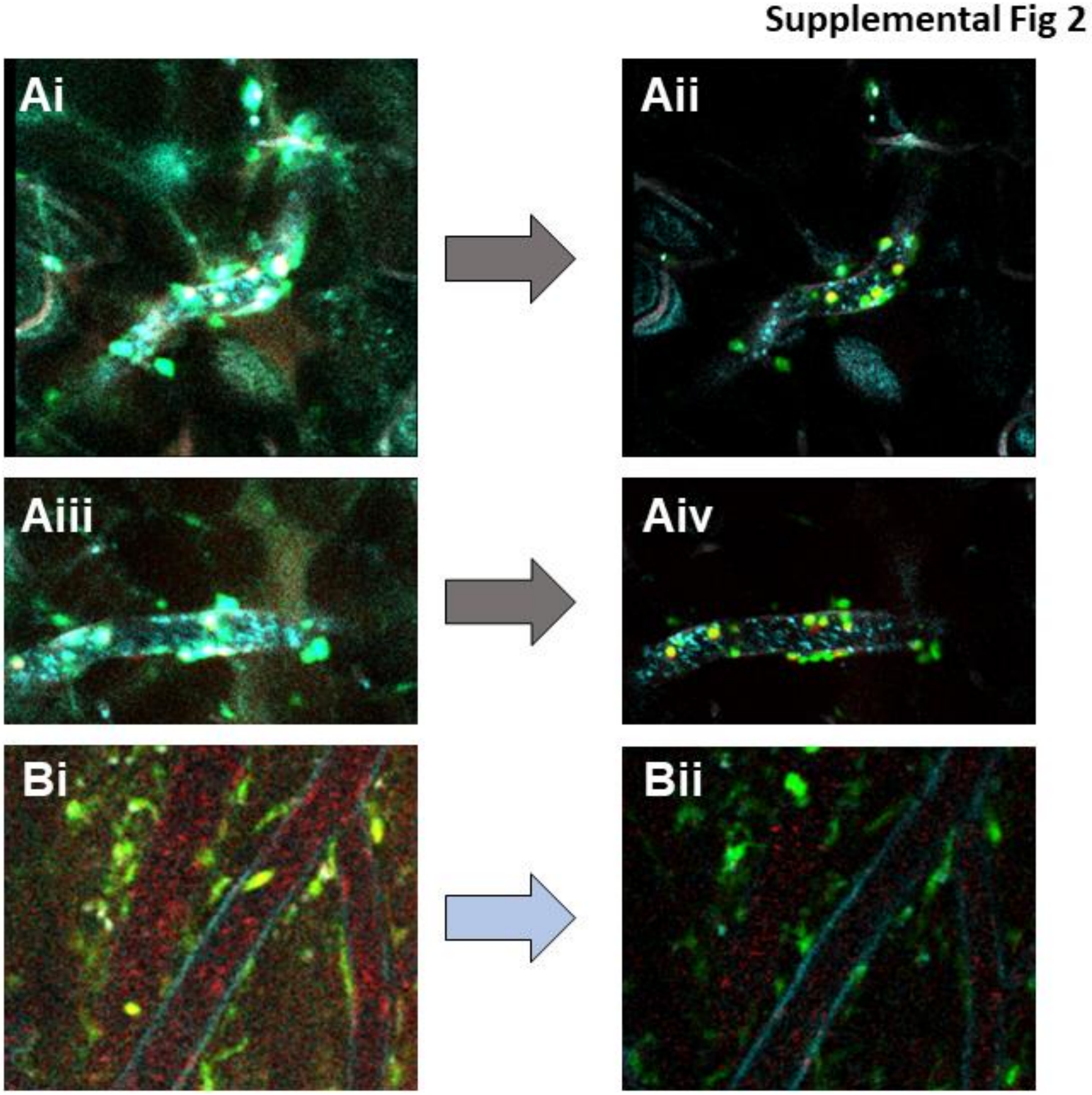

Supplemental Figure 2. Pre- and Post-optimized imaging of other autofluorescent tissues with resonant-scanning confocal intravital microscopy. Skin vasculature was imaged using RSM IVM with emission filters set to mimic a conventional spinning-disk microscopy **(Ai, Aiii)**. Although cells are clearly visible within the vasculature, the wide band-width of emission filters and collecting wavelengths that correspond to peak emission spectra of the labelled antibodies results in substantial spectral bleed-through and prominent autofluorescence making it difficult to visualize and track single cells in these preparations. Optimization of imaging parameters (narrowing detection filters, shifting detectors to collect off-peak fluorescence) dramatically reduces autofluorescence allowing for tracking of not only intravascular leukocytes but also single platelets within the circulation **(Aii, Aiv)**. Fluorescent labels: LysM-GFP (green; neutrophils, monocytes, macrophage), Ly6G (red; neutrophils), CD49b (cyan; platelets), CD31 (gray; endothelium). Imaging of jejunal serosal vasculature with RSM IVM. As described above, tissues were first imaged with emission filters set to mimic a conventional spinning-disk microscopy **(Bi)** followed by imaging with optimized filter settings **(Bii)**. Fluorescent labels: LysM-GFP (green; neutrophils, monocytes, macrophage), CD49b (red; platelets), CD31 (cyan; endothelium).
